# Glucocorticoids suppress early lung inflammation and impair control of SARS-CoV-2 in non-human primates

**DOI:** 10.1371/journal.pone.0342849

**Published:** 2026-03-10

**Authors:** Christine E. Nelson, Keith D. Kauffman, Shunsuke Sakai, Taylor Newbolt, Jay Buchanan, Taylor W. Foreman, April M. Walker, Felipe Gomez, Joel D. Fleegle, Tuberculosis Imaging Program, Richard Herbert, Tom Hill, Sevilay Turan, Katrin D. Mayer-Barber, Reed F. Johnson, Laura E. Via, Daniel L. Barber

**Affiliations:** 1 T Lymphocyte Biology Section, Laboratory of Parasitic Diseases, National Institute of Allergy and Diseases, National Institutes of Health, Bethesda, Maryland, United States of America; 2 Tuberculosis Imaging Program, Division of Intramural Research, National Institute of Allergy and Diseases, National Institutes of Health, Bethesda, Maryland, United States of America; 3 Experimental Primate Virology Section, Comparative Medicine Branch, National Institute of Allergy and Diseases, National Institutes of Health, Bethesda, Maryland, United States of America; 4 Integrated Data Science Section, Research Technologies Branch, National Institute of Allergy and Diseases, National Institutes of Health, Bethesda, Maryland, United States of America; 5 Leidos Biomedical Sciences Inc, Frederick National Laboratory for Cancer Research, National Cancer Institute, Frederick, Maryland, United States of America; 6 Inflammation and Innate Immunity Unit, Laboratory of Clinical Immunology and Microbiology, National Institute of Allergy and Infectious Diseases, National Institutes of Health, Bethesda, Maryland, United States of America; 7 SARS-CoV-2 Virology Core, Laboratory of Viral Diseases, National Institute of Allergy and Infectious Diseases, National Institutes of Health, Bethesda, Maryland, United States of America; 8 Tuberculosis Research Section, Laboratory of Clinical Immunology and Microbiology, Division of Intramural Research, National Institute of Allergy and Infectious Disease, National Institutes of Health, Bethesda, Maryland, United States of America; Rutgers Biomedical and Health Sciences, UNITED STATES OF AMERICA

## Abstract

In severe cases of COVID-19, glucocorticoid treatment improves clinical outcomes. However, in non-hospitalized patients, glucocorticoids have limited benefit and may impair viral clearance. Here, we used the rhesus macaque model of acute SARS-CoV-2 infection to investigate the impact of glucocorticoids on host responses and viral control in a setting of mild disease. Rhesus macaques were pre-treated with intravenous methylprednisolone for 5 days prior to SARS-CoV-2 (Delta) infection and maintained on a daily oral prednisolone until necropsy at day 13 post infection. Glucocorticoid (GC) treatment decreased local lung inflammation measured with ^18^FDG-PET/CT imaging. GC treated animals also had evidence of elevated SARS-CoV-2 viral titers in the lower airways and pulmonary draining lymph nodes. Glucocorticoid treatment blunted plasmacytoid dendritic cell (pDC), eosinophil, gamma delta T cell and early SARS-CoV-2 specific CD8 T cell responses in the airways. These data reveal the cell types that are directly impacted by immunosuppression with glucocorticoids and provide insights into the mechanism of delayed viral clearance observed with glucocorticoid administration during SARS-CoV-2 infection.

## Introduction

Synthetic glucocorticoids (GC) mimic the behavior of naturally occurring glucocorticoids by binding to the glucocorticoid receptor (GCR) and have broad immunosuppressive effects [1–3]. GCs, such as dexamethasone and prednisolone, have been used clinically to treat the most severe cases of COVID-19 [4,5]. In the RECOVERY trial, dexamethasone reduced mortality in COVID-19 patients receiving mechanical ventilation, leading to the treatment recommendation for severe disease [6]. However, GCs are not currently recommended for milder COVID-19 cases. In patients with less severe disease, i.e., those not requiring respiratory support, the RECOVERY trial found dexamethasone had no benefit [6]. Subsequent studies have shown that GCs may actually increase mortality in COVID-19 patients not requiring supplemental oxygen [6–8]. Additionally, individuals taking GCs at the time of SARS-CoV-2 infection can have prolonged viral shedding [9–12]. Collectively the data indicate that GC therapy is beneficial for limiting pathogenic inflammation in severe COVID-19 but may impair resolution of SARS-CoV-2 infection due to suppression of anti-viral immune responses. Thus, there are two major outstanding questions regarding the use of GC during SARS-CoV-2 infection:*1) What is the basis of the therapeutic benefit of GC during severe COVID-19?* and *2) What are the mechanisms underlying impaired viral control during treatment of less severe disease?*

GC treatment has pleiotropic and cell-type specific effects on the immune system [13–16]. Since the 1970s it has been well-documented that GC treatment leads to blood eosinopenia and neutrophilia within 2–4 hours of treatment [17,18]. In a non-human primate model, the reduction in eosinophils after GC treatment was attributed to CXCR4-mediated bone marrow trafficking [19]. GCs can also inhibit dendritic cell activity by dampening antigen-presentation machinery and reducing production of T cell activating cytokines, such as IL-12 [20,21]. GCs have also been shown to inhibit T cell activation by directly suppressing TCR signaling and limiting proliferation [22–24]. GCs also inhibit pro-inflammatory cytokine production by myeloid cells and promote anti-inflammatory, pro-wound healing processes [25,26]. GCs are often used to treat antibody-mediated autoimmune conditions, and GCs have been shown to suppress BCR-dependent activation within two hours of treatment [14]. In severely ill, hospitalized COVID-19 patients plasma concentrations of pro-inflammatory cytokines are reduced with dexamethasone treatment [27–29], and downregulation of an inflammatory gene signature in circulating monocytes with GC treatment is a predictor of survival [30]. However, little is known about the effects of glucocorticoids on early immune responses during milder SARS-CoV-2 infection.

Non-human primates (NHP) have been used extensively to study immunity to SARS-CoV-2 infection and vaccination. Old world NHP, such as rhesus macaques, express a highly similar ortholog of human ACE2 and can be infected with several SARS-CoV-2 variants [31–34]. NHP typically present with mild to moderate disease, depending on the age and species of NHP [35–40]. SARS-CoV-2 infected NHP have evidence of local lung inflammation, including edema, alveolar thickening, type-II pneumocyte hyperplasia, and cellular infiltration that ultimately resolve [36,39,40]. NHP therefore serve as a model of milder SARS-CoV-2 infection observed in most individuals. While macaques are not appropriate for studying the effects of GC treatment during severe COVID-19 disease, they may be useful for investigating the effects of GC treatment during less severe disease and can provide mechanistic insights into the immunological causes of impaired control of SARS-CoV-2 infection observed with GC treatment.

Here, we infected GC-treated rhesus macaques with SARS-CoV-2 to investigate the effects of GCs on the cellular immune response in the airways and determine the impact on viral control. Animals were pre-treated with high dose intravenous (i.v.) methylprednisolone, infected with SARS-CoV-2 Delta variant, and maintained on prednisolone until necropsy at day 13 post-infection. Lung inflammation was assessed with ^18^FDG PET/CT imaging, viral replication was evaluated in the upper and lower respiratory tract, and single cell RNA sequencing and spectral flow cytometry was performed on immune samples collected from the blood and airways. GC treated animals had evidence of prolonged viral replication and elevated peak viral titers in the lung. This was associated with reduced ^18^FDG uptake in the lungs, as well as decreased eosinophils, plasmacytoid DCs, gamma delta T cells, and SARS-CoV-2 specific T cells in the airways. These data suggest that GCs delay clearance of SARS-CoV-2 infection from the lower respiratory tract, which may be attributable to reduced early innate immune responses to SARS-CoV-2 infection in the airways.

## Results

A pilot study was performed to examine the effects of GC treatment in the rhesus macaque model (S1A Fig). Two male rhesus macaques received a priming regimen of 4 mg/kg intravenous methylprednisolone daily for five days, followed by maintenance dosing of daily oral prednisolone at 0.5 mg/kg for an additional 13 days. At two hours post treatment, there was a drop in transcripts for *TNF* and *IL1B* and an increase in glucocorticoid induced-leucine zipper (GLIZ) *TSC22D3*, in the blood and bronchoalveolar lavage (BAL) (S1B and S1F Fig). *TNF* and *IL1B* expression levels in the blood remained elevated at day 2 but returned to baseline by day 4 after treatment. In the blood, GC treatment caused a transient increase in neutrophils and a concomitant decrease in eosinophils, monocytes, T cells, and NK cells, at two hours post-treatment, which is consistent with patient data after GC treatment (S1C–E Fig) [14,17–19]. The effects of GC treatment on the BAL immune composition were more variable and delayed compared to the response in the blood (S1G–I Fig). Thus, GC treatment in rhesus macaques is associated with dynamic but transient changes in immune landscape in the circulation and airways that return to homeostatic levels within one week of continuous treatment.

**Fig 1:**
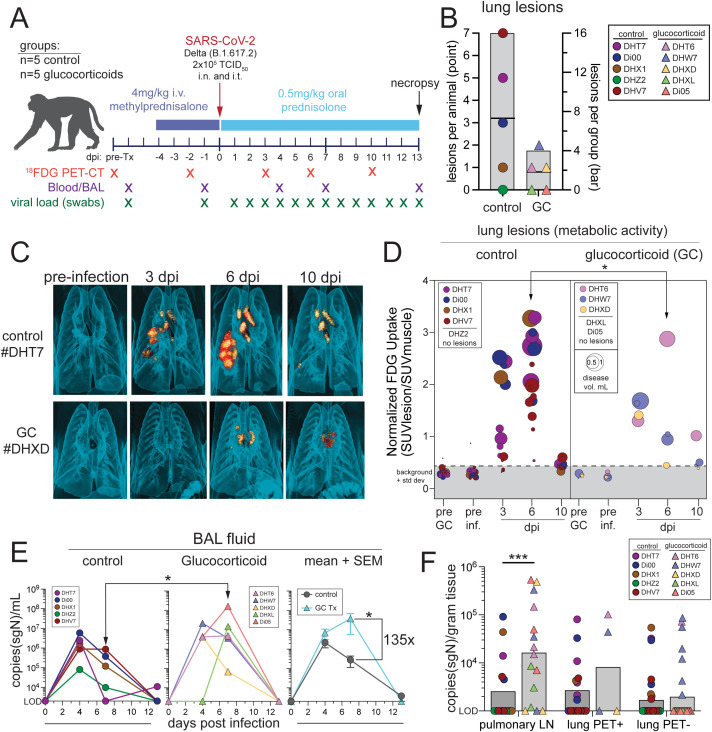
Glucocorticoid treatment suppresses SARS-CoV-2 induced lung inflammation and increases viral replication in the lower airways. A) Experimental design and sample collection. Created in part with BioRender under CC BY license, Nelson, C., (2025). B) Quantification of total lung lesions detected with ^18^FDG-PET/CT imaging at the peak of the response, per animal and per group with mean. Animal #ID represented by color and shape in legend. C) Example ^18^FDG-PET/CT images from control animal #DGT7 and GC treated animal #DHXD. D) Quantification of the lung lesion volume (dot size) and the metabolic intensity (normalized FDG uptake = SUV of lesion/SUV muscle). Significance calculated with 2way Anova multiple comparison test at the indicated timepoints between GC treatment and control. E) Quantification of subgenomic RNA of the SARS-CoV-2 N1 protein in copies per mL of BAL fluid. Individual animals and the mean of each group with standard error mean represented. Significance calculated with 2way Anova. Fold change between GC treatment and control indicated. Limit of detection (LOD) is 2,000 copies/mL fluid. F) Subgenomic N1 in copies per gram of tissue in the pulmonary lymph nodes, PET+ involved lung, and PET- uninvolved lung. Significance calculated with 2way Anova multiple comparison test. LOD is 1,000 copies per gram of tissue. p > 0.05 not shown, *p < 0.05, ***p < 0.001.

To investigate the effects of GC during SARS-CoV-2 infection, male rhesus macaques were treated with GC at the dosing established in the pilot study or given saline as a control, with n = 5 subjects in each group (Fig 1A). After 5 days of treatment, all animals were infected with SARS-CoV-2 Delta variant (B.1.617.2). The Delta variant is more virulent in rhesus macaques compared to other SARS-CoV-2 variants [41,42]. GC treated animals were maintained on prednisolone until necropsy at day 13 after infection. ^18^FDG-PET/CT imaging was performed to assess for local lung inflammation. We previously showed that FDG-avid lung inflammation peaks 3−6 days after infection with SARS-CoV-2/USA/WA1 isolate [39,43]. With SARS-CoV-2 Delta infection, 4 of 5 animals in the control group had detectable lung lesions, with 1–7 lesions per animal, consistent with SARS-CoV-2 USA/WA-1, but lung inflammation peaked at 6 days post infection with the Delta strain (Fig 1B–D) [39,43]. GC treatment reduced the number, intensity, and duration of FDG-avid lung lesions (Fig 1B–D). GC treated animals had fewer than 2 lesions each, and 2 of 5 had no detectable lesions (Fig 1B). The lesions that were present in the GC treated animals also resolved faster than in control animals (Fig 1D). Thus, glucocorticoids can suppress early lung inflammation induced by acute SARS-CoV-2 infection.

Subgenomic viral RNA was measured in nasal swabs, oral swabs, and BAL throughout infection, and in tissues at necropsy (Fig 1A). In the control group, nucleocapsid viral RNA in the BAL peaked at day 4 after infection (Fig 1E). At day 7, viral RNA loads were ~135-fold higher in GC treated animals than in saline controls (Fig 1E). Four of 5 GC treated animal had detectable live SARS-CoV-2 virus in the BAL at day 7 post-infection, as measured by TCID_50_ assay, compared to 2 of 5 in the control group (S2A Fig). At day 13 necropsy, viral RNA loads were higher in the pulmonary draining lymph nodes of GC treated animals compared to controls, consistent with increased viral replication in the airways (Fig 1F). There was no difference in viral RNA detected in lung tissue samples (Fig 1F), nasal or oral swabs, nasopharynx, nasal turbinates, tonsils, olfactory bulb, brain stem, frontal cortex, cerebrospinal fluid (CSF), cervical lymph nodes, axillary lymph nodes, or spleen (S2B–D Fig). These data suggest that immunosuppression with GC led to increased viral replication in the lower respiratory tract during the first week of SARS-CoV-2 Delta infection. These data are consistent with previous studies showing increased MERS-CoV viral shedding in NHP treated with dexamethasone [44].

**Fig 2:**
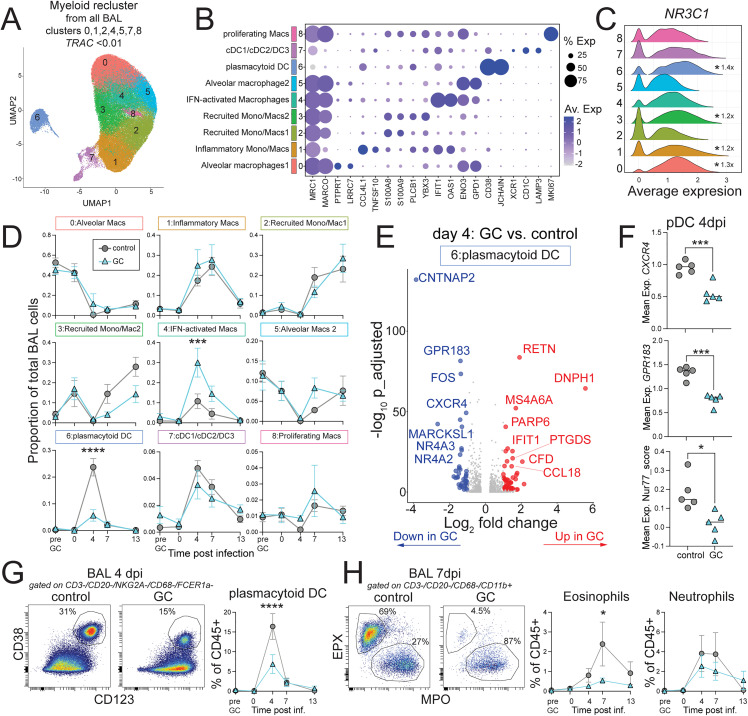
Glucocorticoid treatment dampens plasmacytoid DC and eosinophil accumulation in the airways. A) Unsupervised clustering and UMAP projection of myeloid/DC cell subsets (from total BAL clusters 0,1,2,4,5,7,8) with TRAC<0.01. B) Selected top differentially expressed genes in each myeloid cell cluster from *A*. Size of the dot represents % expressed and the color scale is the average expression. C) Normalized expression of *NR3C1* in each myeloid cluster. D) The proportion of myeloid clusters 0-8 of total BAL cells in GC treatment and control, with the group mean and SEM indicated. Significance calculated with 2-way Anova and multiple comparison test. E) Differentially expressed genes in plasmacytoid DC (pDC) at day 4 post-infection between GC treatment vs. control. |Log_2_ fold-change| > 1 and adjusted p-value < 0.05 are highlighted. Red is upregulated in GC treated with log_2_FC > 1 and blue is downregulated with log_2_FC < −1 compared to control. Grey is ns or absolute |log_2_FC| < 1. F) Mean expression of *CXCR4*, *GPR183*, and Nur77_score (combined *NR4A1*, *NR4A2, NR4A3*) in pDC day 4 post infection, for each animal. Significance calculated with unpaired t-test. G) Representative flow cytometry of pDC (CD38 + /CD123+) in BAL at day 4 post-infection, gated on live/CD45 + /CD3-/CD20-/NKG2A-/CD68-/FceR1a- and quantification of % pDC of total BAL cells that are CD45 + , overtime. Significance calculated with 2-way anova and multiple comparison test. H) Representative flow cytometry of eosinophils (EPX + /MPO-) and neutrophils (MPO + /EPX-) in BAL at day 4 post-infection, gated on live/CD45 + /CD3-/CD20-/CD68-/CD11b+ and quantification of % of total BAL CD45 + , overtime. Significance calculated with 2-way Anova and multiple comparison test. p > 0.05 not shown, *p < 0.05, ***p < 0.001. ****p < 0.0001.

To determine which immune subsets are affected by GC treatment single cell RNA sequencing was carried out on BAL samples from pre-treatment, day 0, 4, 7, and 13 post-SARS-CoV-2 infection (Fig 1A). Several populations of innate and adaptive immune cells were identified in the BAL, including myeloid/DC cells (clusters 0,1,2,4,5,7,8), T cells (clusters 3 and 10), B cells (cluster 9), Mast cells (cluster 6), and epithelial cells (cluster 11) (S3A, B Fig). A subset analysis of the myeloid/DC cells was performed (Figs 2A, B and S3A–C). All myeloid cells expressed the GC receptor (*NR3C1*), with alveolar macrophages (myeloid cluster 0), inflammatory monocyte/macrophages (myeloid cluster 1), recruited monocyte-derived macrophages (myeloid cluster 3), and plasmacytoid DCs (myeloid cluster 6) having the highest expression of the GC receptor (Fig 2C). BAL cell numbers recovered were similar between GC treatment and controls throughout the study (S4A Fig). At baseline, alveolar macrophages were the dominant cell type in the BAL (myeloid clusters 0,5) (Fig 2D). The BAL composition was similar between GC treatment and control groups at the time of SARS-CoV-2 infection, after 5 days of i.v. methylprednisolone treatment, which is consistent with the results from the pilot study where cellular changes induced by GC treatment had returned to homeostatic levels within the first week of treatment (S1 Fig). At day 4 after SARS-CoV-2 infection, there was a predictable loss of alveolar macrophages (clusters 0 and 5) in both groups, and an influx of inflammatory macrophages (cluster 1) and IFN-activated macrophages (cluster 4), as has been shown previously [39]. There was greater abundance of IFN-activated macrophages in the GC treated group, consistent with increased viral replication in the BAL (Fig 2D). Expression of *IFNB1* was found exclusively in inflammatory macrophages and GC treatment led to a lower overall peak but prolonged *IFNB1* response after infection (S3D, E Fig)*.* Several myeloid cell clusters in GC treated animals had marginally elevated expression of interferon stimulated genes between days 4 and 13 post-infection, which was measured with an interferon stimulated gene (ISG) score including 22 ISGs (S3F, G Fig). Differential expression analysis of day 4 IFN-activated macrophages showed downregulation of ISGs and increased *IL1B* with GC treatment (S3H Fig). IL-1β is inversely correlated with type I IFN expression in other model systems [45,46]. Together these data suggest that increased SARS-CoV-2 viral replication observed with GC treatment resulted in dysregulated type I IFN responses in airway myeloid cell populations.

In both groups, there was an influx of dendritic cells in the BAL at day 4 after SARS-CoV-2 infection, which included plasmacytoid DCs (myeloid cluster 6) and a mixture of conventional DC subsets (myeloid cluster 7) (Fig 2B, D). The day 4 plasmacytoid DC (pDC) response was significantly dampened by GC treatment (Fig 2D). pDCs from GC treated animals had lower expression of *CXCR4* and *GPR183* compared to controls (Fig 2E, F). CXCR4 downregulation following GC treatment may be a result of CXCR4-mediated homing to the bone marrow, as has been shown for eosinophils [19]. GPR183 has also been implicated in myeloid cell lung homing [47]. pDCs from GC treated animals had diminished expression of immediate-early response genes in the Nur77 family, i.e., *NR4A1*, *NR4A2*, and *NR4A3* (Fig 2E, F)*.* Nur77 expression is important in pDC differentiation [48,49]. The reduced pDC responses in the BAL with GC treatment was confirmed by flow cytometry (Fig 2G). Granulocytes are not well-represented in this single cell RNA sequencing datasets due to cryopreservation methods used. However, flow cytometry revealed an influx of neutrophils and eosinophils into the airways that peaked at day 4−7 after infection (Fig 2H). GC treatment significantly dampened the eosinophil response but had no effect on neutrophil recruitment (Fig 2H). The reduction of eosinophils with GC treatment was temporally correlated with the increase in viral loads observed after GC treatment. Thus, GC treatment dampens pDC and eosinophil recruitment to the airways in response to SARS-CoV-2 infection leading to increased viral replication and increased type I IFN responses in some myeloid populations.

**Fig 3:**
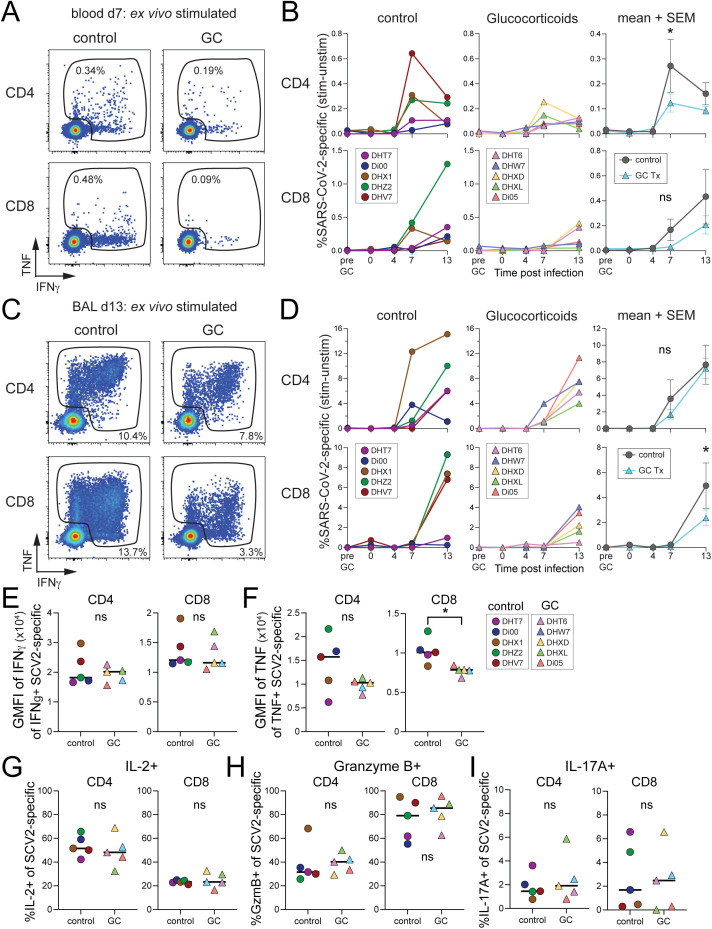
Glucocorticoid treatment dampens peptide-specific T cell responses to SARS-CoV-2 infection in the airways. A) Representative flow cytometry of SARS-CoV-2 specific CD4 and CD8 T cell responses (IFNγ + /TNF+) in blood at day 7 post-infection after *ex vivo* peptide stimulation with Spike and Nucleocapsid peptide pools, gated on live/CD45 + /CD3 + /CD95 + . B) Quantification of the frequency of SARS-CoV-2 specific CD4 and CD8 T cell responses in the blood overtime after subtracting background staining in unstimulated samples. Significance calculated with 2-way Anova and multiple comparison test. C) Representative flow cytometry of SARS-CoV-2 specific CD4 and CD8 T cell responses (IFNγ + /TNF+) in BAL at day 13 post-infection after *ex vivo* peptide stimulation with Spike and Nucleocapsid peptide pools, gated on live/CD45 + /CD3 + /CD95 + . D) Quantification of the frequency of SARS-CoV-2 specific CD4 and CD8 T cell responses in the BAL overtime after subtracting background staining in unstimulated samples. Significance calculated with 2-way Anova and multiple comparison test. E) Geometric mean fluorescence intensity (GMFI) of IFNγ production by IFNγ + SARS-CoV-2 specific CD4 and CD8 T cells. F) GMFI of TNF production by TNF+ SARS-CoV-2 specific CD4 and CD8 T cells. G) Frequency of IL-2+ of SARS-CoV-2 specific (IFNγ + /TNF+) CD4 and CD8 T cells in the BAL at day 13 post-infection. Significance calculated with unpaired t-test. H) Frequency of Granzyme B+ of SARS-CoV-2 specific (IFNγ + /TNF+) CD4 and CD8 T cells in the BAL at day 13 post-infection. I) Frequency of IL-17A+ of SARS-CoV-2 specific (IFNγ + /TNF+) CD4 and CD8 T cells in the BAL at day 13 post-infection. p > 0.05 ns or not shown, *p < 0.05, ***p < 0.001. ****p < 0.000.

In rhesus macaques, SARS-CoV-2-specific T cells can be detected as early as 7 days post infection in the BAL and continue to increase in frequency for at least 4−5 weeks after initial infection [43]. Antigen-specific CD4 and CD8 T cell response were assessed by *ex vivo* peptide re-stimulation with a mixture of peptides pools derived from spike (B.1.617.2) and nucleocapsid proteins. In the blood, SARS-CoV-2-specific CD4 T cell responses peaked at day 7 after infection and were reduced with GC treatment (Fig 3A, B). There were no differences in the early induction of the Ag-specific CD8 T cells in the blood. In the BAL, there was a slight reduction in frequency of SARS-CoV-2-specific CD8 T cells in the GC treatment group and a reduction in the geometric mean fluorescence intensity of (GMFI) of TNF staining among SARS-CoV-2-specific CD8 T cells (Fig 3C–F). There were no differences in the production of IL-2, IL-17A, or Granzyme B among SARS-CoV-2-specific CD4 or CD8 T cells in BAL (Fig 3G–I). There were also no differences in the frequency of regulatory T cells (Tregs) or NK cells, as has been reported previously for GC treatment (S4C, D) [50,51]. Thus, GC treatment had a minor impact on the magnitude of the SARS-CoV-2 specific T cell response early after infection. It is unclear how these differences would impact long-term maintenance of SARS-CoV-2-specific T cell memory.

**Fig 4:**
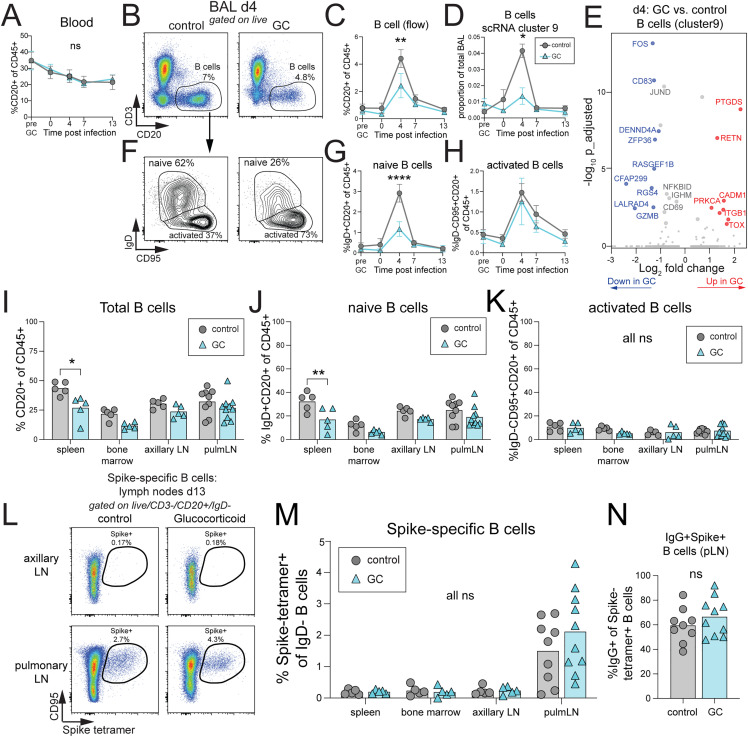
Glucocorticoid treatment results in a reduction in naïve-like B cell recruitment to the lungs after SARS-CoV-2 infection. A) Quantification of total B cells (CD3^-^/CD20^+^) by flow cytometry in the blood over time. Mean of each group with standard error mean (SEM) represented. Significance calculated with 2way Anova. B) Representative flow cytometry plots and gating strategy for identifying total B cells (CD3^-^/CD20^+^) from the BAL at day 4 after infection. C) Quantification of total B cells by flow cytometry in the BAL over time. Mean, SEM, and 2way Anova. D) Quantification of total B cells by scRNAseq (cluster 9) in the BAL over time. Mean, SEM, and 2way Anova. E) Differentially expressed genes in B cells (cluster 9) at day 4 post-infection between GC treatment vs. control. Red is upregulated with GC treatment, log_2_FC > 1 and adjusted p-value <0.05. Blue is downregulated with GC treatment, log_2_FC < −1 and adjusted p-value <0.05. Grey with large dot is adjusted p-value <0.05 but absolute |log_2_FC| < 1. Grey with small dot is adjusted p-value >0.05. F) Representative flow cytometry plots and gating strategy for identifying naïve B cells (CD3^-^/CD20^+^/IgD^+^) and activated B cell (CD3^-^/CD20^+^/IgD^-^/CD95^+^) from the BAL at day 4 after infection. G) Quantification of naïve B cells by flow cytometry in the BAL over time. Mean, SEM, and 2way Anova. H) Quantification of activated B cells by flow cytometry in the BAL over time. Mean, SEM, and 2way Anova. I) Quantification of total B cells, J) naïve B cells, K) and activated B cells in the spleen, bone marrow, axillary lymph nodes, and pulmonary lymph nodes at necropsy day 13 post-infection. Significance calculated with 2way Anova. L) Representative flow cytometry plots of Spike-specific B cells (CD3^-^/CD20^+^/CD95^+^/Spike-tetramer^+^) isolated from the axillary or pulmonary lymph nodes at necropsy, day 13 post-infection. M) Quantification of Spike-specific B cells (CD3^-^/CD20^+^/CD95^+^/Spike (B.1.617.2) tetramer^+^) as a percentage of non-naïve (IgD^-^) B cells in the spleen, bone marrow, axillary lymph nodes, and pulmonary lymph nodes at necropsy day 13 post-infection. N) Quantification of the frequency of IgG^+^ expression by Spike-specific (tetramer^+^) B cells in the pulmonary lymph node. Significance calculated with 2way Anova. p > 0.05 ns or not shown, *p < 0.05, ***p < 0.001. ****p < 0.0001.

In rhesus macaques, Spike-specific antibody responses can be detected as early as 2 weeks post SARS-CoV-2 infection, and titers continue to increase 4−5 weeks after infection [43]. Early B cell responses in the blood and BAL were evaluated after GC treatment and infection. While there were no changes in the frequency of total B cells in the blood, very early B cell recruitment to the lungs at day 4 post-infection was significantly decreased in the GC treatment group, as measured by flow cytometry and single cell RNA sequencing (Fig 4A–D). This decrease in B cell recruitment to the airways was largely due to reduced influx of naïve-like, IgD^+^ B cells to the lungs that peaked at day 4 after SARS-CoV- 2 infection (Fig 4E–H). Differential gene expression of B cells from the BAL at day 4 after infection showed a reduction in B cell activation after GC treatment, with significantly reduced *FOS*, *CD83*, and *IGHM* expression (Fig 4E). While the decrease in *IGHM* was significant, it did not reach the threshold of greater than 2-fold change compared to control B cells. The reduction in naïve B cell recruitment induced by SARS-CoV-2 infection with GC treatment fits with previously published reports of reduced IgM^+^ B cells in the circulation at day 2 after GC treatment [14]. At day 13 necropsy, there was also a reduction in total B cells in the spleen, that was also attributable to a reduced naïve IgD^+^ B cell response (Fig 4I–K). Spike-specific B cell responses were evaluated in the spleen, bone marrow, axillary lymph nodes, and pulmonary draining lymph nodes using B cell tetramers loaded with spike protein from B.1.617.2 (Delta) variant. Early spike-specific B cells responses were exclusively detected in the pulmonary draining lymph nodes and not found in other lymphoid organs (Fig 4L, M). There was no difference between treatment groups in the frequency of Spike-specific B cells or activation status, as measured by IgG^+^ class-switching (Fig 4L–N). Therefore, GC treatment reduced the very early recruitment of naïve-like B cells to the airways at day 4 after SARS-CoV-2 infection, but this did not directly impact induction of early Spike-specific B cells. However, these data cannot not address the role of GC treatment in the long-term maintenance of SARS-CoV-2-specific B cell memory.

**Fig 5:**
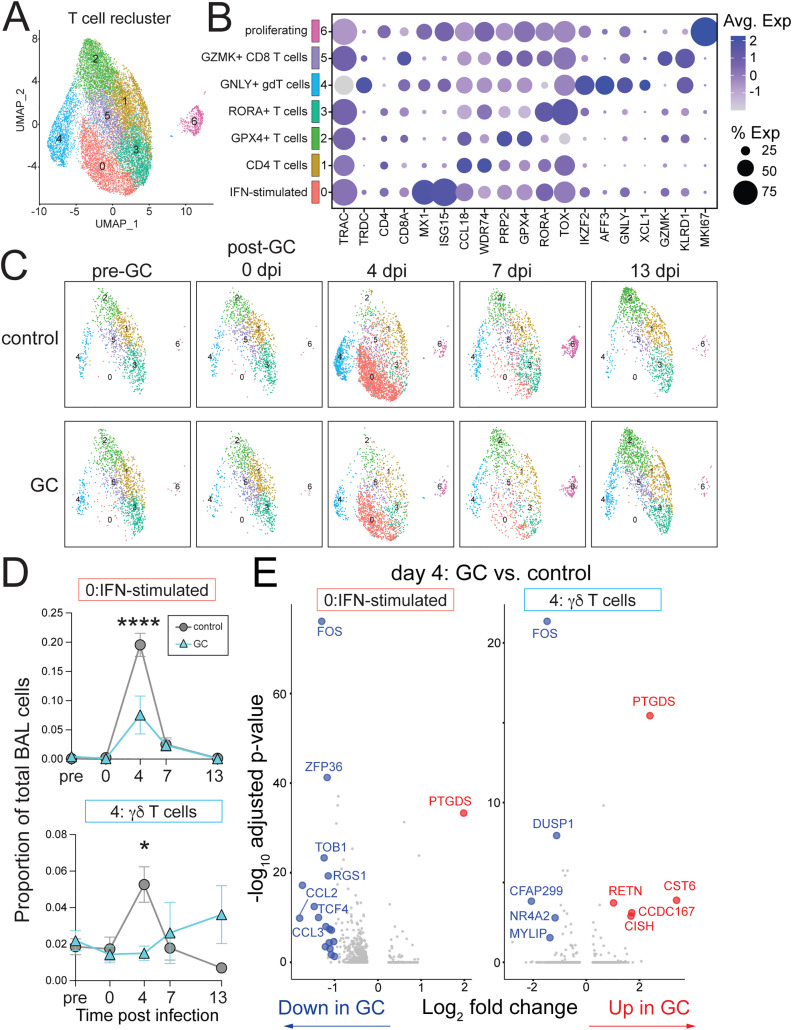
Glucocorticoid treatment inhibits γδ T cell responses to SARS-CoV-2 infection in the airways. A) Unsupervised clustering and UMAP projection of scRNAseq and re-clustering of T cells (from total BAL cluster 3,10). B) Selected top differentially expressed genes in each T cell cluster. Size of the dot represents % expressed and the color scale the average expression. C) UMAP as in *A*, separated by timepoint. D) Proportion of IFN-stimulated T cells (cluster 0) and gamma delta (γδ) T cells (cluster 4) of total BAL cells. Significance calculated with 2-way Anova and multiple comparison test. E) Differentially expressed genes in IFN-stimulated T cells (cluster 0) and γδT cells (cluster 4) at day 4 post-infection between GC treatment vs. control. Log_2_ fold-change > 1 and adjusted p-value < 0.05 are highlighted. Red is upregulated in GC treated with log_2_FC > 1 and blue is downregulated with log_2_FC < −1 compared to control. Grey is ns or absolute |log_2_FC| < 1. *FOS, NR4A2, and PTGDS* are bolded. p > 0.05 ns or not shown, *p < 0.05, ****p < 0.0001.

To further evaluate T cell responses to GC treatment and SARS-CoV-2 infection, a subset analysis of *TRAC*+ T cells from scRNAseq was performed. A mixture of innate-like and adaptive CD4+ and CD8 + T cells subsets were identified. The early response to SARS-CoV-2 in BAL at day 4 after infection was dominated by an influx of IFN-stimulated T cells (T cell cluster 0) and granulysin positive (*GNLY*+) gamma delta (γδ) T cells (T cell cluster 4) that also had high expression of *IKZF2*, *AFF3,* and *XCL1* (Fig 5A–D). Both IFN-stimulated and γδ T cell responses to infection were significantly blunted by GC treatment (Fig 5C, D). Differential gene expression of IFN-stimulated and γδ T cells showed a downregulation of immediate early response genes, including *NR4A2* and *FOS*, and an increase in prostaglandin D2 synthase, *PTGDS*, in GC treated versus control (Fig 5E). While glucocorticoid treatment has been shown to inhibit prostaglandins, increases in *PTGDS* gene expression and PGDS enzyme activity have been observed in mouse neuronal and kidney cells after dexamethasone treatment [52,53]. These data suggest that the largest effect of glucocorticoid treatment on T cell responses during SARS-CoV-2 infection are on a population of *TRDC*+ T cells in the airways.

## Discussion

This study investigated the cell-type specific effects of glucocorticoids during acute SARS-CoV-2 infection of a resistant host. A small pilot study of the glucocorticoid treatment regimen in uninfected rhesus macaques demonstrated a rapid drop in eosinophils, monocytes, T cells, B cells, and NK cells with a corresponding increase in neutrophils in the blood within the first two hours, similar to what has been previously reported [14,17–19]. In the bronchoalveolar lavage (BAL), the cell type specific changes were more variable and delayed compared to the blood. Importantly, most of the initial immune perturbations triggered by GC treatment had returned to baseline levels within ~5 days in blood and BAL, and no further changes to immune cell compositions were seen after the day 5 switch to oral GC treatment. Therefore, day 5 was chosen to start SARS-CoV-2 infection in subsequent studies where the effects of glucocorticoids on the host immune response and viral control were assessed.

GC treatment during SARS-CoV-2 infection led to prolonged viral replication and higher peak titers in the lower respiratory tract on day 7 after infection. On day 4 post infection, GC treatment reduces the early influx of plasmacytoid DCs (pDC), gamma delta (γδ) T cells and naïve B cells into the airways. GC treatment also significantly blunts eosinophil responses in the airways at day 7, which temporally correlates with prolonged viral replication and increases in peak viral titers in the airways. Although not typically thought of as anti-viral cells, eosinophils have been shown to mediate antiviral defense in pulmonary viral infections and have been implicated in the enhanced control of SARS-CoV-2 infection in asthmatics [54–56]. Future studies are needed to directly test the contribution of each cell type to the increased viral replication with GC treatment.

Type I IFN is critical for control of SARS-CoV-2 infection. Individuals with diminished type I IFN responses are more likely to have severe COVID-19 disease [57,58]. Monocytes and macrophages are among the first cell types to respond to type I IFN in the BAL after SARS-CoV-2 in rhesus macaques [39]. Interestingly, GC treatment increased IFN-driven gene expression in macrophages after SARS-CoV-2 infection. pDCs can be major producers of type I IFN but were overall reduced with GC treatment during SARS-CoV-2 infection. Type I IFN (*IFNB1*) in the BAL was primarily expressed by inflammatory macrophages, and *IFNB1* expression by macrophages was delayed but prolonged with GC treatment compared to controls. The increase in IFN-stimulated macrophages and prolonged type I IFN response in the GC treated animals may reflect an ongoing response to infection and fits with the delayed viral clearance observed in the respiratory tract. In contrast to myeloid cells, GC treatment reduced the type I IFN activation of T cells in the BAL. In fact, the largest change in the composition of T cells at day 4 post infection with GC treatment was the loss of IFN-stimulated CD4 and CD8 T cells. Therefore, during SARS-CoV-2 infection GC treatment alters the kinetics of IFNβ1 expression by macrophages and differentially effects type I IFN induced gene expression in myeloid populations and T cells.

GC treatment slightly reduced the magnitude of SARS-CoV-2 specific T cells in the blood and BAL. One limitation of this study is that it did not extend beyond day 13 post-infection. A previous study showed that the durability of SARS-CoV-2 specific T cell responses in the blood from individuals receiving GC treatment was dependent on the dose and duration of treatment [59]. More studies are needed to understand how GC impact maintenance of SARS-CoV-2-specific tissue resident memory T cells in the respiratory tract.

GC treatment also dampened the early influx of γδT cells in the BAL after SARS-CoV-2 infection, suggesting these T cells could also play an important role in the early anti-viral immune response. Increases in circulating γδT cells have been reported in SARS-CoV-2 infection and after vaccination [60,61]. In the BAL, γδT cells were expanded at day 4 after infection in control animals and expressed high levels of granulysin (*GNLY*) and the chemokine *XCL1*. XCL1 is a marker of tissue resident CD8 T cells and a potent recruiter of XCR1 + cDC1s [62,63]. Future studies are needed to better understand the role of these γδT cell responses in SARS-CoV-2.

This study utilized rhesus macaques, which are a well-established model of acute SARS-CoV-2 infection. This model is ideal for understanding the immune modulatory effects of glucocorticoids leading to impaired clearance of SARS-CoV-2 infection in a resistant host. However, this model cannot be used to understand the effects of GC treatment in severe COVID-19. A limitation of this study is that the mechanisms leading to impaired viral control with GC treatment during mild SARS-CoV-2 disease may differ from mechanisms of GC-mediated immune suppression that benefit severely ill patients. Therefore, more work is needed to develop animal models that can accurately replicate the clinical outcomes of GC treatment along with dosing and timing used clinically for severe COVID-19 cases. Additionally, this study utilized the SARS-CoV-2 delta variant, and it is not clear if GC treatment of animals infected with currently circulating strains would differ from the results here. Nonetheless, our data show that GC treatment during SARS-CoV-2 infection may impair viral control by suppressing the early influx of eosinophils, pDCs, and innate-like T and B cell responses into the airways.

## Materials and methods

### Animal study design

#### Rhesus macaques.

Male Indian-origin rhesus macaques (*Macaca mulatta*) were sourced from the National Institute of Allergy and Infectious Diseases breeding colony on Morgan Island, South Carolina. Animals were maintained in accordance with the Animal Welfare Act, the Guide for the Care and Use of Laboratory Animals, and all applicable regulations, standards, and policies. Animals were singly housed in nonhuman primate biocontainment racks with 12-hour light/dark cycle, at 60–70°C, and relative humidity 30–70% in a fully Association for Assessment and Accreditation of Laboratory Animal Care (AAALAC) International–accredited Animal Biosafety Level 3 (ABSL3) vivarium at the NIH. Animals were fed a high fiber commercial diet that contains 20% protein twice a day and supplemented with primate biscuits. Water was offered ad libitum. Animals were provided with a variety of enrichment including toys, treats, fresh produce, and foraging devices. Procedures were conducted as outlined in the NIAID Division of Intramural Research Animal Care and Use Committee-approved Animal Study Proposal LPD-25E. For all procedures, animals were sedated with ketamine (4–10 mg/kg) and dexmedetomidine (0.03 mg/kg) intramuscularly and closely monitored for heart rate, respiratory rate, body temperature, and oxygen saturation throughout the procedure. Glycopyrrolate (0.01 mg/kg) and Atipamezole (250–300ug/kg) was given intramuscularly to reverse anesthesia. Animals were given fruit and soft food during recovery from anesthesia. Animals were monitored twice daily for clinical signs, including general appearance, appetite, water consumption, activity, skin and hair coat, condition of mucosal surfaces, respiration, stool and urine production and quality. No analgesia was administered for study symptoms. Euthanasia of the research animals was performed in accordance with the American Veterinary Medical Association Guidelines. For euthanasia, animals are sedated as indicated above and an overdose of Beuthanasia phenytoin/pentobarbital solution at 1mL per 10lbs is delivered intravenously. Death confirmed with absence of heartbeat/pulse and respiration along with pupil dilation and absence of corneal reflex.

#### Study design.

(*Pilot experiment*) Two male rhesus macaques were administered methylprednisolone at 4 mg/kg intravenous (i.v.) daily for 5 days and then maintained on a 0.5 mg/kg dose of oral prednisolone daily for 13 days. (*Infection experiment*) Ten male rhesus macaques, aged 2–3 years, were divided into glucocorticoid (GC) and control (saline) treatment groups, n = 5 each. Methylprednisolone was administered at 4 mg/kg i.v. daily for 5 days and then maintained with 0.5 mg/kg dose of oral prednisolone daily until day 13 post infection necropsy. Animals were infected with 2x10^5^ TCID_50_ of SARS-CoV-2 Delta variant (B.1.617.2) via intranasal and intratracheal routes at day 5 of methylprednisolone treatment. Experiments were approved by the NIAID ACUC under animal safety protocol LPD-25E in an AAALAC accredited aBSL-3 vivarium facility.

FDG-PET/CT Analysis: Animals were sedated and on mechanical ventilation for PET/CT imaging. [^18^F]-FDG dose of 0.5 mCi/kg was given intravenously 1 hour prior to PET imaging. High-resolution CT scan of the lungs was acquired with a breath hold on a LFER 150 PET/CT scanner (Mediso Inc.) as previously described [39]. Data were reconstructed using the Nucline software (Mediso, Inc.) to create individual DICOM files that were co-registered using MIM Maestro (v. 7.0, MIM Software Inc.). For each animal, the lesion VOIs at the peak of the response were transferred to all the other timepoints by aligning the PET/CT images. Values were extracted from the original and transferred VOIs. Two readers independently performed image analysis. Three-dimensional projections of FDG uptake in the lung regions were generated using Osirix v 5.9 software (Pixmeo).

#### Sample collection.

Animals were anesthetized with ketamine and dexmedetomidine for i.v. injections, PET/CT scans, viral swabs, blood and BAL collection. Glycopyrrolate and Atipamezole were given for recovery from anesthesia. Whole blood was collected in EDTA tubes and cryopreserved in Cryostor CS10 media (StemCell Cat#07930) at 1:10 dilution. BAL was collected after intubation by instillation of 50mL of warm pharmaceutical-grade PBS, 10mLs at a time. BAL was filtered through a 100um filter into a 50mL conical and centrifuged at 1,600 rpm for 15 min at 4°C. The cell pellet was cryopreserved in Cryostor CS10 media. Swabs were collected in 1mL viral transport media (1x HBSS, 2% FBS, 100ug/mL Gentamicin, and 0.5ug/mL amphotericin B). Intravenous antibody was administered prior to euthanasia, as previously described [64]. PET/CT guided prosection of the lung and airways was performed, as previously described, to isolate previously PET+ and PET- lung sections [39,43]. Nasopharynx, nasal turbinates, salivary gland, tonsils, olfactory bulb, frontal cortex, brain stem, spleen, cervical lymph node, and axillary lymph nodes were also collected.

### Viral quantification methods

#### RT-qPCR.

RNA was extracted from 140uL of sample (BAL or swab media), processed using a Viral RNA mini kit (Qiagen Cat# 52906), and eluted in 50uL RNase Free water. Tissue pieces were stored in RNAlater (Sigma Cat# R0901) at 4°C overnight and then −80°C until RNA extraction. Thawed tissues were processed in the RNeasy Plus Mini kit (Qiagen # 74136) and eluted in 50uL. RNA from whole blood was extracted with QIAamp RNA Blood Mini Kit (Qiagen Cat#) and eluted in 50uL. Extracted RNA was prepared in a 12.5uL RT-qPCR reaction, with 2.5uL of eluted RNA, 3.25uL Taqpath 1-step RT-qPCR Master Mix (Thermo Cat#A15299), primers at 500nM, probes at 125-200nM, and the remaining volume as RNase free water.

N1 gene subgenomic RNA was detected as previously described [39,43]. RNA integrity was verified using the 2019-nCoV RUO Kit for RNase P. Copies per/mL, copies/gram, and limit of detection were calculated based on SARS-CoV-2 RNA standard curve. *IL1B* was detected using Forward primer (5’-TGGCATCCAGCTACAAATCTC-3’), Reverse (5’-CCAGCATCTTCCTTAGCTTCTC-3’), and Probe (5’-FAM/CACGAGCACTACAACGAGGGCTTC/3IABkFQ/-3’). *TNF* was detected using Forward primer (5’-CATCTACTCCCAGGTCCTCTT-3’), Reverse (5’- TTGACCTTGGTCTGGTAGGA-3’), and Probe (5’-FAM/ ATGTGCTCCTCACCCACACCATC/3IABkFQ/-3’). *TS22D3* was detected using Forward primer (5’-CAAGCCAGTGAGCCTCTAAT-3’), Reverse (5’-CACACCTCAAACTACCCTTCTC-3’), and Probe (5’-FAM/TTTGCCAGT TGCAGCTAAGTTGCC/3IABkFQ/-3’). *ACTB* was detected using Forward primer (5’-GGATCAGCAAGCAGGAGTATG-3’), Reverse (5’- AGAAAGGGTGTAACGCAACTAA-3’), and Probe (5’-FAM/TCGTCCACCGCAAATGCTTCTAGG/3IABkFQ/-3’). Cycling conditions: Initial: 25°C for 2 min, 50°C for 15 min, and 95°C for 3 min, Cycling: 95°C for 5s, 58.5°C for 30s, for 40 cycles. Fold change was calculated by normalizing to *ACTB*. 2^ΔΔ^ = 2^[t_0_ CT(*target*)-t_0_ CT(*ATCB*))- (t_n_CT(*target*)- (t_n_CT(*ACTB*))]. All primers and probes custom made from Eurofins. Reactions were read on a QuantStudio 7 Flex Real-Time PCR System (Applied Biosystems).

#### TCID_50_ assay.

2x10^4^ *TMPRSS2 +*Vero E6 cells were plated in a flat bottom 96 well plate. The following day media was removed, and 200uL of samples were added. 12, 5-fold serial dilutions of BAL samples were plated, with 4-replicates each. Plates were incubated for 4 days at 37°C with 5% CO_2_. Media was removed and 50uL of 0.5% Crytal Violet solution (25% CV (Sigma Cat#HT90132), 20% Ethanol, 55% water) was added and incubated for 10 minutes. CV was removed and 100uL of sterile water was added. Liquid was removed and plates were allowed to dry. Plates were scored for CPE. TCID_50_ was calculated with the Spearman & Karber algorithm.

### Immune cell quantification

#### Peptide stimulation assay.

Cells were thawed, and single cell suspensions were plated at 2x10^7^ cell/mL in 200uL with X-VIVO 15 media, plus 10% FBS. Cells were rested overnight at 37°C + 5% CO_2_ then washed and stimulated with peptides plus Brefeldin A 1000x (Invitrogen Cat#00-4506-51) and Monensin 1000x (Invitrogen Cat#00-4505-51) for 6 hours at 37°C + 5% CO_2_ before surface staining. Peptide pool consisted of Peptivator SARS-CoV-2 Prot_S Complete (Miltenyi Cat#130-127-953), Peptivator SARS-CoV-2 Prot_S B.1.617.2 Mutation (Miltenyi Cat#130-128-763), and Peptivator SARS-CoV-2 Prot_N (Miltenyi Cat# 130-126-699). After stimulation cells were washed and proceeded with surface staining. The frequency of antigen-specific T cells, IFNγ+ or TNF + CD4+ or CD8 + , was adjusted for background staining in unstimulated wells (% stimulated - % unstimulated).

#### Flow cytometry.

Cells were resuspended in 100uL of live dead NIR 780 (Thermo Cat#L34994), diluted 1:1000 in PBS and incubated for 15 minutes. Cells were washed and stained with 50uL surface stain antibodies diluted in PBS + 1% FBS and incubated for 20 min at 4°C. Cells were washed 3 times with PBS + 1% FBS, before fixation with eBioscience Intracellular Fixation & Permeabilization Buffer Set (Thermo Cat# 88-8824-00) for 16 hours at 4°C. After fixation cells were washed with eBioscience Permeabilization Buffer, resuspended in 50uL intracellular stains diluted in Perm. Buffer, and stained for 30 min at 4°C. After staining cells were washed with Perm. Buffer 2x and resuspended in 180uL of PBS + 1% FBS + 0.05% Sodium Azide for analysis on the Cytek Aurora 5L.

B cell tetramers were prepared by combining Recombinant SARS-CoV-2 Spike-Prot B.1.617.2 (HEK) Biotin (Miltenyi cat#130-129-565) at 0.1 mg/mL with streptavidin BV605 at 50ug/mL and PEB buffer (PBS + 0.45% BSA, 2mM EDTA) for 15 minutes prior to staining. B cell tetramer staining was performed between live dead and surface staining and incubated for 30 minutes at room temperature.

### Single cell RNA sequencing

Cryopreserved BAL cells were processed for scRNAseq using the 10X Genomics Chromium Single Cell 3′ kit (v3.1). Samples were then stained with unique TotalSeq-A hashtag antibodies (HTO) as per manufacturer’s (Biolegend) protocol. Equal number of cells from each sample were pooled and super-loaded on a 10X Genomics Next GEM chip and single cell GEMs were generated on a 10X Chromium Controller. Subsequent steps to generate cDNA and HTO libraries were performed following 10X Genomics and Biolegend’s protocol respectively. Libraries were pooled and sequenced on an Illumina NovaSeq. Sequenced data was processed using cellranger (version 7.1.0) to demultiplex the libraries. The reads were aligned to mmul_10 genome to generate count tables. Sample batch effects were corrected using Harmony R package (version 1.2.3). The count tables were then further processed and analyzed using the Seurat (version 5.2.1) in R (version 4.4.1).

#### Statistical analysis.

Data were analyzed using a two-way ANOVA with a Sidak’s multiple comparison test or standard t-test. Tests used are indicated in figure legends. For all statistical analysis p < 0.05 for the given test is considered significant: * p < 0.05, ** p < 0.01, *** p < 0.001, **** p < 0.0001.

## Supporting information

S1 Fig Glucocorticoid treatment causes transient neutrophilia, eosinopenia, lymphopenia in the blood and BAL of rhesus macaques. A) Experimental design and sample collection. Created in part with BioRender under CC BY license, Nelson, C., (2025). B) Fold change in *TNF*, *IL1B*, and *TSC22D3* RNA transcripts in whole blood at the indicated timepoints after treatment measured by qRT-PCR, normalized to *ACTB*. 2^ΔΔ^ = 2^[t_0_ CT(*target*)-t_0_ CT(*ATCB*))- (t_n_CT(*target*)- (t_n_CT(*ACTB*))]. C) Unsupervised clustering and UMAP projection of concatenated flow cytometry from blood at all timepoints, pre-gated on live/CD45 + cells, with the indicated populations: Monocytes (HLA-DR^+^/CD11b^+^), B cells (CD3^-^/CD20^+^), CD8 T cells (CD20^-^/CD3^+^/CD8a^+^/CD8b^+^/CD4^-^), CD4 T cells (CD20^-^/CD3^+^/CD4^+^/CD8a^-^), Neutrophils (CD68^-^/HLA-DR^-^/CD11b^+^/CD66abce^+^/EPX^-/^MPO^+^), Eosinophils (CD68^-^/HLA-DR^-^/CD11b^+^/CD66abce^+^/ MPO^-^/EPX^+^), gamma-delta T cells (CD3^+^/γδTCR^+^), NK cells (CD3^-^/CD20^-^/NKG2A^+^/CD8b^-^), plasmacytoid DC (CD68^-^/HLA-DR^+^/CD14-/CD16^-^/FceR1a^-^/CD123^+^), naïve B cells (CD3^-^/CD20^+^/IgD^+^), activated B cells (CD3^-^/CD20^+^/IgD^-^/IgG^+/-^), naïve T cells (CD20^-^/CD3^+^/CD4^+/-^/CD8a^+/-^/Foxp3^-^/CD28^+^/CD95^-^), memory CD4 T cells (CD20^-^/CD3^+^/CD4^+/-^/CD8a^+/-^/Foxp3^-^/CD28^+^/CD95^+^), terminal effectors (CD20^-^/CD3^+^/CD4^+/-^/CD8a^+/-^/Foxp3^-^/CD28^-^/CD95^+^). D) UMAP as in *C*, separated by timepoint. E) Quantification of Neutrophils, Eosinophils, Monocytes, T cells, B cells, and NK cells as a percentage of live/CD45 + cells in whole blood after GC treatment. F) Fold change in *TNF*, *IL1B*, and *TSCD22D3* RNA transcripts in BAL at the indicated timepoints after treatment measured by qRT-PCR, normalized to *ACTB*. G) Unsupervised clustering and UMAP projection of concatenated flow cytometry from BAL at all timepoints, pre-gated on live/CD45 + cells, with the indicated populations. Gating strategy as in *C*. H) UMAP as in *G*, separated by timepoint. I) Quantification of Neutrophils, Alveolar Macrophages, Monocytes, T cells, B cells, and NK cells as a percentage of live/CD45 + cells in BAL after GC treatment. Statistics not included due to small sample size and low statistical power.
(TIF)

S2 Fig Glucocorticoids do not impact viral replication in the nasal and oral swabs or in other tissues at necropsy. A) Quantification of replication competent SARS-CoV-2 per mL of BAL fluid measured by TCID_50_ assay with Vero/TMPRSS2 cells. Individual animals and the mean of each group with standard error mean represented. Significance calculated with 2way Anova. Limit of detection (LOD) is 1 copy/mL. B) Quantification of subgenomic RNA of the SARS-CoV-2 N1 protein in copies per mL of nasal and oral swab fluid. Individual animals and the mean of each group with standard error mean represented. Significance calculated with 2way Anova. Limit of detection (LOD) is 2,000copies/mL. C) Subgenomic N1 in copies per gram of tissue in the nasopharynx, nasal turbinates, tonsils, salivary gland, olfactory bulb, brain stem, frontal cortex, cervical lymph node, axillary lymph node, and spleen. Significance calculated with 2way Anova multiple comparison test. LOD is 1,000 copies per gram of tissue. D) Subgenomic RNA of the SARS-CoV-2 N1 protein in copies per mL of cerebrospinal fluid (CSF). Limit of detection (LOD) is 2,000copies/mL. p > 0.05 not shown, *p < 0.05.
(TIF)

S3 Fig Glucocorticoid treatment leads to prolonged type I IFN responses after SARS-CoV-2 infection. A) Unsupervised clustering and UMAP projection of total BAL cells from scRNAseq from all samples and all timepoints. B) Selected top differentially expressed genes in each cell cluster from *A*. Size of the dot represents % expressed and the color scale is the average expression. C) Re-clustering of myeloid cells from total BAL clusters 0,1,2,4,5,7,8 with TRAC<0.01. D) Normalized average expression of *IFNB1* expression by myeloid cells, separated by timepoint and treatment condition. E) Percent *IFNB1*+ (Expression. > 0.1) of cluster 1, inflammatory macrophages, over time. F) *Left*, Genes included in ISG_score. *Right,* Normalized average expression of ISG_score in myeloid cell clusters defined in *B*, separated by timepoint and treatment condition. G) Quantification of mean ISG_Score for each myeloid cluster overtime. Significance calculated with 2way Anova multiple comparison test. Red stars = significantly higher in GC treated. Blue stars = significantly down in GC treated. Significance calculated with 2way Anova multiple comparison test. *p < 0.05. H) Differentially expressed genes in myeloid cluster 4, IFN-activated macrophages, at day 4 after infection, between GC treatment vs. control. Log_2_ fold-change > 1 and adjusted p-value < 0.05 are highlighted. Red is upregulated in GC treated with log_2_FC > 1 and blue is downregulated with log_2_FC < −1 compared to control. Grey is ns or absolute |log_2_FC| < 1.
(TIF)

S4 Fig Glucocorticoid treatment did not alter the frequency of Tregs or NK cells. A) Quantification of the total live cell count recovered from the BAL wash at each timepoint. Mean of each group with standard deviation. All ns p < 0.05. Significance calculated with 2-way Anova. B) Total live cells isolated from the indicated tissues. Significance calculated with 2way Anova. C) Quantification of total T regulatory cells (Tregs) (CD3^+^/CD4^+^/Foxp3^+^) of total CD45^+^ cell in the blood and BAL measured by flow cytometry over time. Mean of each group with standard error mean (SEM) represented. Significance calculated with 2way Anova. D) Quantification of total NK cells (CD3^-^/CD8α^+^/CD8β^-^/NKG2A^+^) of total CD45^+^ cell in the blood and BAL measured by flow cytometry over time. Mean of each group with standard error mean (SEM) represented. Significance calculated with 2way Anova. p > 0.05 is non-significant (ns).
(TIF)
