# Supplementary figures and images for: Glucocorticoids suppress early lung inflammation and impair control of SARS-CoV-2 in non-human primates
Source: PLoS One. 2026 Mar 10;21(3):e0342849. doi: 10.1371/journal.pone.0342849 (PMC12974840; doi:10.1371/journal.pone.0342849)

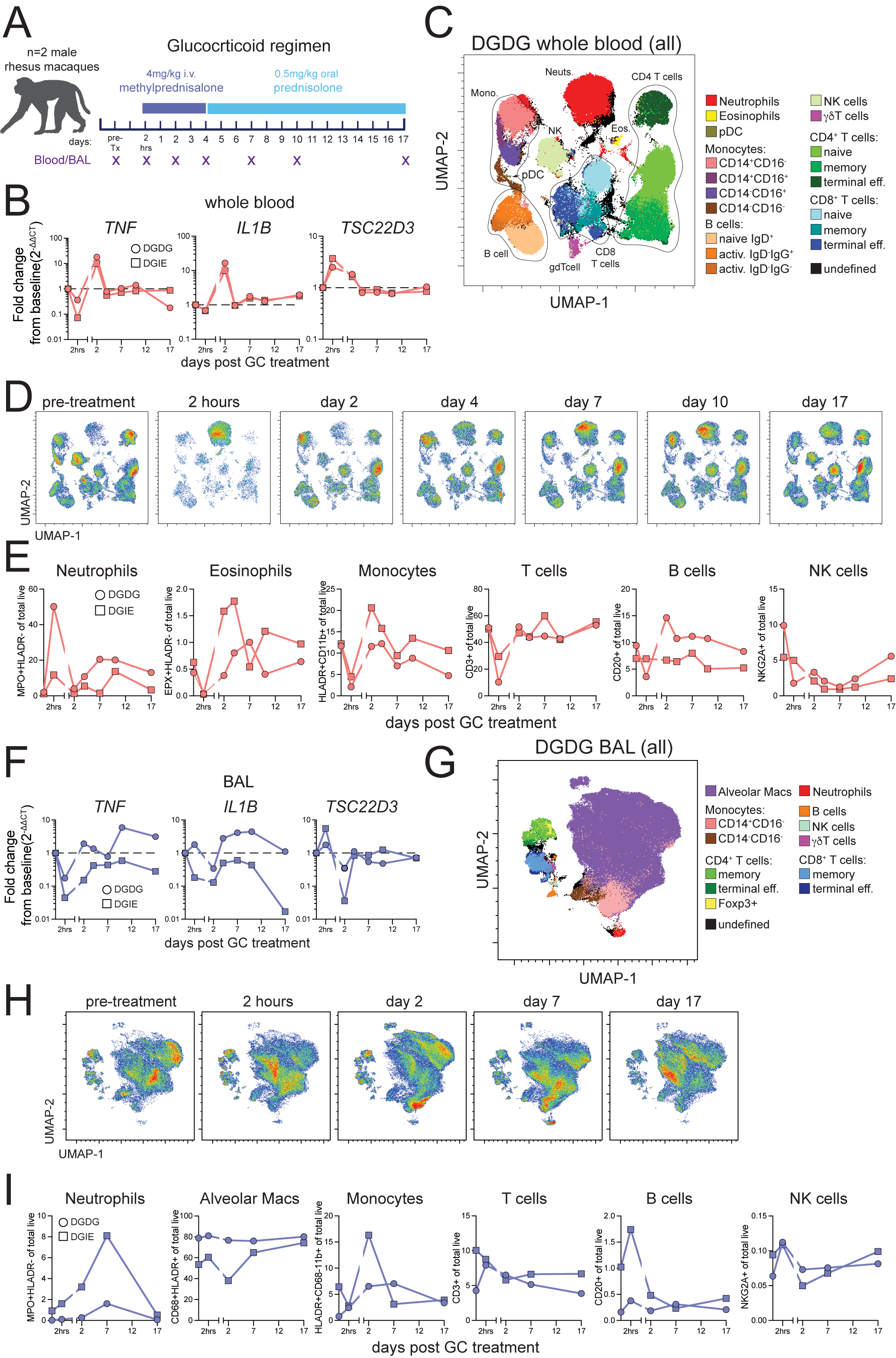

Supplement: S1 Fig — A) Experimental design and sample collection. Created in part with BioRender under CC BY license, Nelson, C., (2025). B) Fold change in TNF, IL1B, and TSC22D3 RNA transcripts in whole blood at the indicated timepoints after treatment measured by qRT-PCR, normalized to ACTB. 2ΔΔ = 2^[t0 CT(target)-t0 CT(ATCB))- (tnCT(target)- (tnCT(ACTB))]. C) Unsupervised clustering and UMAP projection of concatenated flow cytometry from blood at all timepoints, pre-gated on live/CD45 + cells, with the indicated populations: Monocytes (HLA-DR+/CD11b+), B cells (CD3-/CD20+), CD8 T cells (CD20-/CD3+/CD8a+/CD8b+/CD4-), CD4 T cells (CD20-/CD3+/CD4+/CD8a-), Neutrophils (CD68-/HLA-DR-/CD11b+/CD66abce+/EPX-/MPO+), Eosinophils (CD68-/HLA-DR-/CD11b+/CD66abce+/ MPO-/EPX+), gamma-delta T cells (CD3+/γδTCR+), NK cells (CD3-/CD20-/NKG2A+/CD8b-), plasmacytoid DC (CD68-/HLA-DR+/CD14-/CD16-/FceR1a-/CD123+), naïve B cells (CD3-/CD20+/IgD+), activated B cells (CD3-/CD20+/IgD-/IgG+/-), naïve T cells (CD20-/CD3+/CD4+/-/CD8a+/-/Foxp3-/CD28+/CD95-), memory CD4 T cells (CD20-/CD3+/CD4+/-/CD8a+/-/Foxp3-/CD28+/CD95+), terminal effectors (CD20-/CD3+/CD4+/-/CD8a+/-/Foxp3-/CD28-/CD95+). D) UMAP as in C, separated by timepoint. E) Quantification of Neutrophils, Eosinophils, Monocytes, T cells, B cells, and NK cells as a percentage of live/CD45 + cells in whole blood after GC treatment. F) Fold change in TNF, IL1B, and TSCD22D3 RNA transcripts in BAL at the indicated timepoints after treatment measured by qRT-PCR, normalized to ACTB. G) Unsupervised clustering and UMAP projection of concatenated flow cytometry from BAL at all timepoints, pre-gated on live/CD45 + cells, with the indicated populations. Gating strategy as in C. H) UMAP as in G, separated by timepoint. I) Quantification of Neutrophils, Alveolar Macrophages, Monocytes, T cells, B cells, and NK cells as a percentage of live/CD45 + cells in BAL after GC treatment. Statistics not included due to small sample size and low statistical power. (TIF) [file pone.0342849.s001.tif]

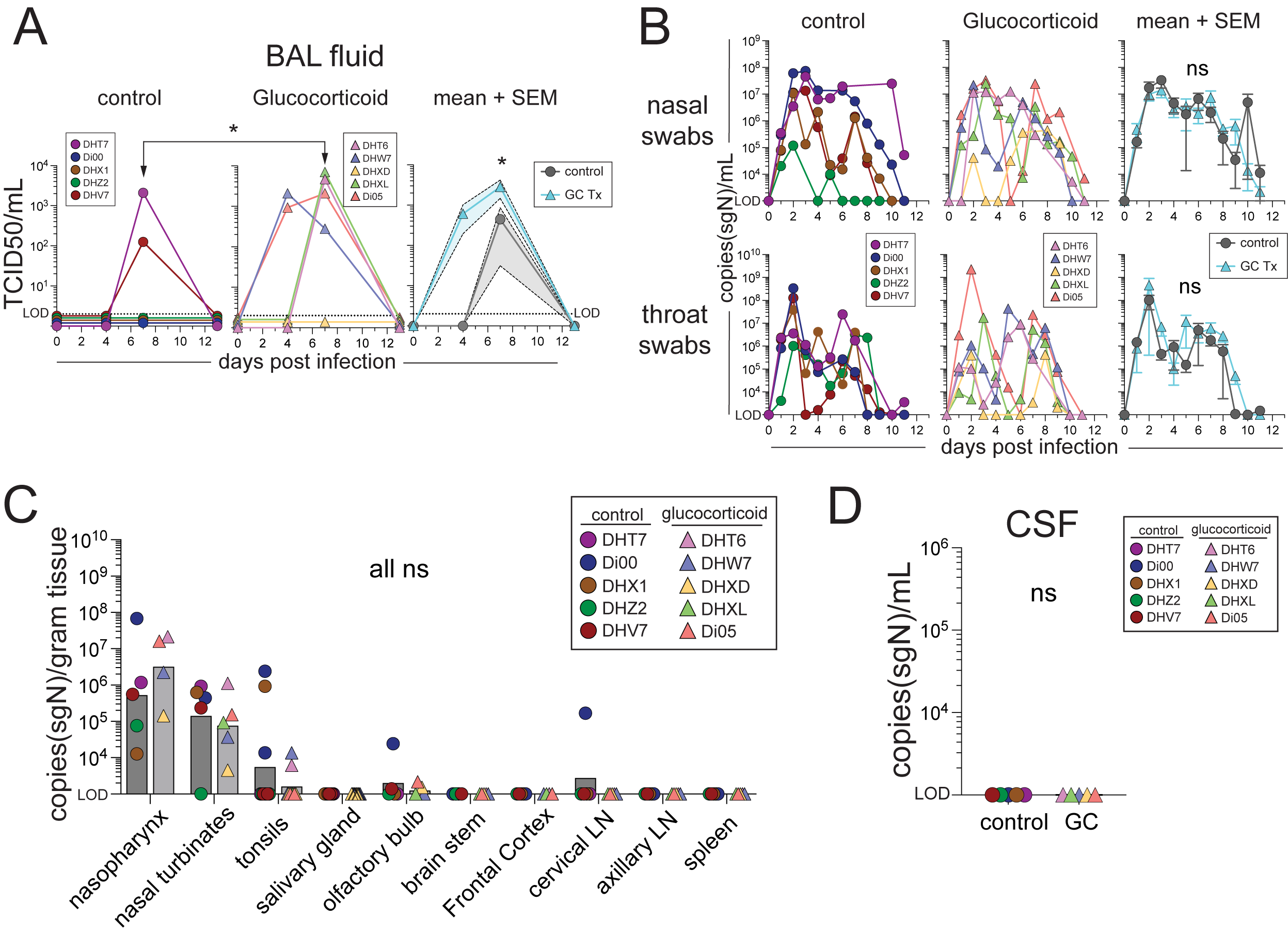

Supplement: S2 Fig — A) Quantification of replication competent SARS-CoV-2 per mL of BAL fluid measured by TCID50 assay with Vero/TMPRSS2 cells. Individual animals and the mean of each group with standard error mean represented. Significance calculated with 2way Anova. Limit of detection (LOD) is 1 copy/mL. B) Quantification of subgenomic RNA of the SARS-CoV-2 N1 protein in copies per mL of nasal and oral swab fluid. Individual animals and the mean of each group with standard error mean represented. Significance calculated with 2way Anova. Limit of detection (LOD) is 2,000copies/mL. C) Subgenomic N1 in copies per gram of tissue in the nasopharynx, nasal turbinates, tonsils, salivary gland, olfactory bulb, brain stem, frontal cortex, cervical lymph node, axillary lymph node, and spleen. Significance calculated with 2way Anova multiple comparison test. LOD is 1,000 copies per gram of tissue. D) Subgenomic RNA of the SARS-CoV-2 N1 protein in copies per mL of cerebrospinal fluid (CSF). Limit of detection (LOD) is 2,000copies/mL. p > 0.05 not shown, *p < 0.05. (TIF) [file pone.0342849.s002.tif]

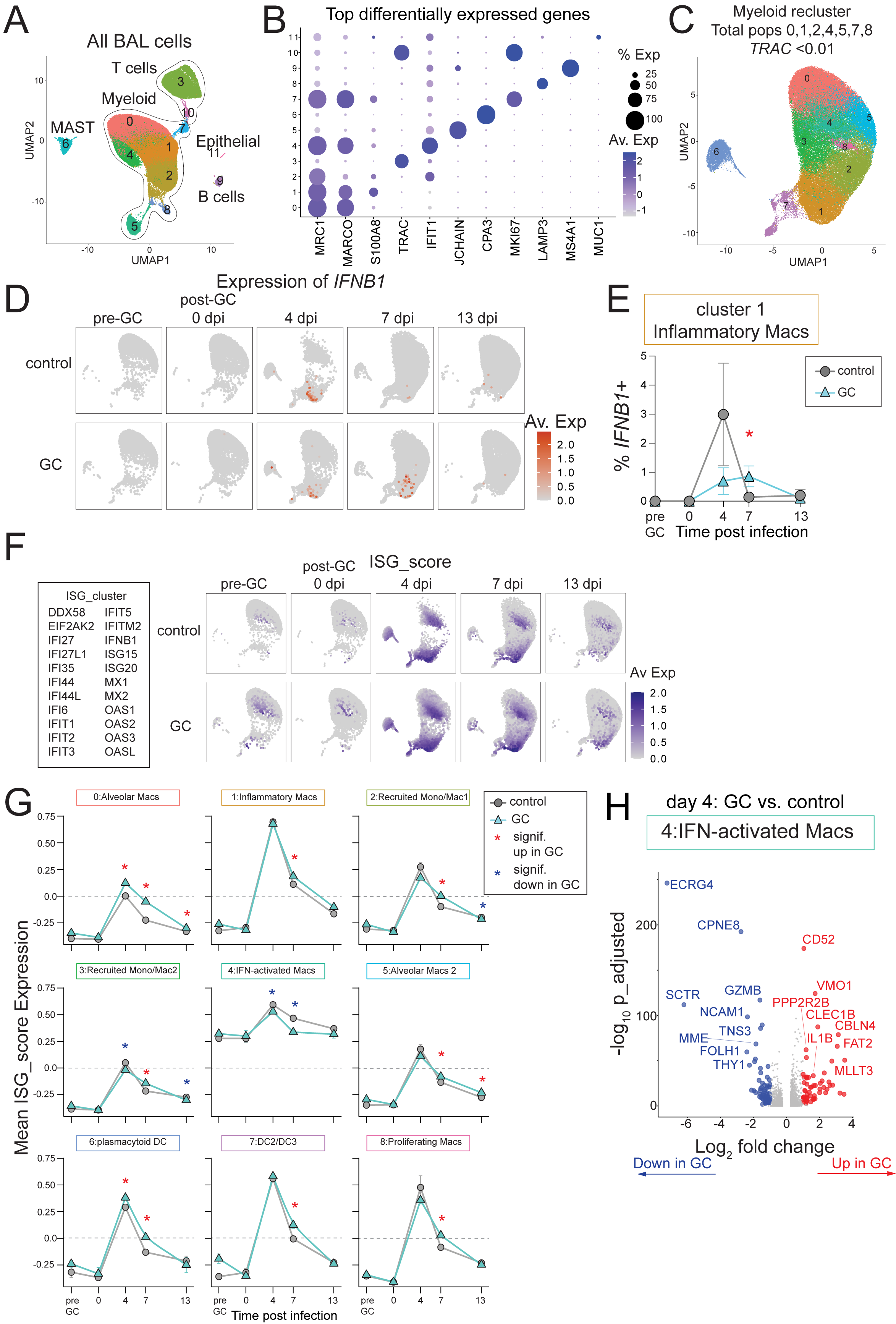

Supplement: S3 Fig — A) Unsupervised clustering and UMAP projection of total BAL cells from scRNAseq from all samples and all timepoints. B) Selected top differentially expressed genes in each cell cluster from A. Size of the dot represents % expressed and the color scale is the average expression. C) Re-clustering of myeloid cells from total BAL clusters 0,1,2,4,5,7,8 with TRAC<0.01. D) Normalized average expression of IFNB1 expression by myeloid cells, separated by timepoint and treatment condition. E) Percent IFNB1+ (Expression. > 0.1) of cluster 1, inflammatory macrophages, over time. F) Left, Genes included in ISG_score. Right, Normalized average expression of ISG_score in myeloid cell clusters defined in B, separated by timepoint and treatment condition. G) Quantification of mean ISG_Score for each myeloid cluster overtime. Significance calculated with 2way Anova multiple comparison test. Red stars = significantly higher in GC treated. Blue stars = significantly down in GC treated. Significance calculated with 2way Anova multiple comparison test. *p < 0.05. H) Differentially expressed genes in myeloid cluster 4, IFN-activated macrophages, at day 4 after infection, between GC treatment vs. control. Log2 fold-change > 1 and adjusted p-value < 0.05 are highlighted. Red is upregulated in GC treated with log2FC > 1 and blue is downregulated with log2FC < −1 compared to control. Grey is ns or absolute |log2FC| < 1. (TIF) [file pone.0342849.s003.tif]

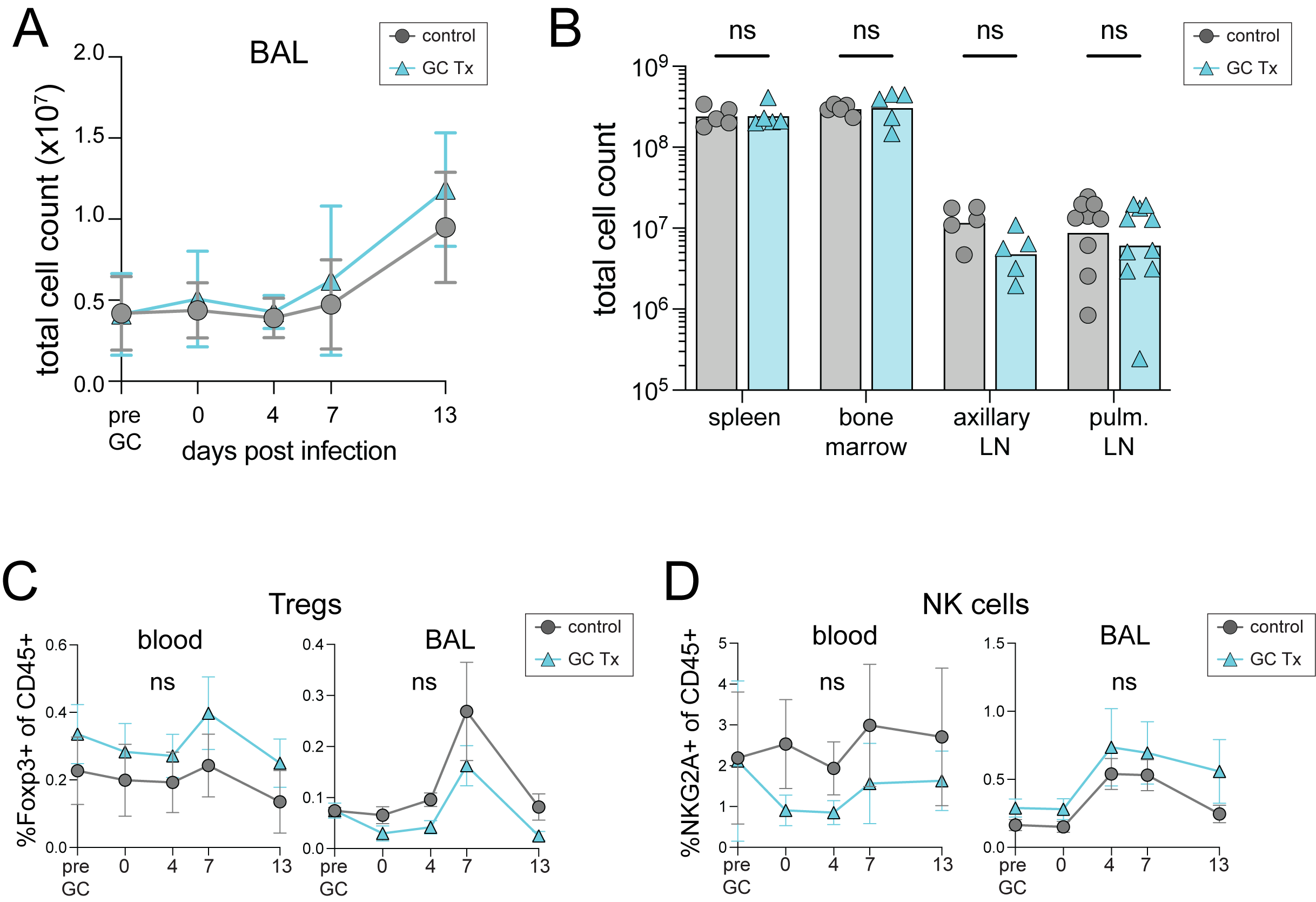

Supplement: S4 Fig — A) Quantification of the total live cell count recovered from the BAL wash at each timepoint. Mean of each group with standard deviation. All ns p < 0.05. Significance calculated with 2-way Anova. B) Total live cells isolated from the indicated tissues. Significance calculated with 2way Anova. C) Quantification of total T regulatory cells (Tregs) (CD3+/CD4+/Foxp3+) of total CD45+ cell in the blood and BAL measured by flow cytometry over time. Mean of each group with standard error mean (SEM) represented. Significance calculated with 2way Anova. D) Quantification of total NK cells (CD3-/CD8α+/CD8β-/NKG2A+) of total CD45+ cell in the blood and BAL measured by flow cytometry over time. Mean of each group with standard error mean (SEM) represented. Significance calculated with 2way Anova. p > 0.05 is non-significant (ns). (TIF) [file pone.0342849.s004.tif]
